# *Lactobacillus plantarum 17-5* Alleviates *Escherichia coli* Mastitis by Inhibiting the cGAS-STING Pathway

**DOI:** 10.3390/ani15223305

**Published:** 2025-11-16

**Authors:** Jia-Ze Han, Meng-Meng Li, Xiao-Wen Yu, Rui-Ning Zhang, Qian Zou, Jun-Chi Deng, Fa-Jian Zhao, Han-Qing Li, Ke Li, Zhen-Gui Yan

**Affiliations:** 1 College of Veterinary Medicine, Shandong Agricultural University, 7 Panhe Street, Tai’an 271017, China; m18649110585@163.com (J.-Z.H.); 18753410122@163.com (M.-M.L.); 13173302926@163.com (X.-W.Y.); 18366561500@163.com (R.-N.Z.); zrsm0618@163.com (Q.Z.); djcwyyx2021@163.com (J.-C.D.); m13295371016@163.com (F.-J.Z.); lihanq@sdau.edu.cn (H.-Q.L.); 2 Shandong Provincial Key Laboratory of Zoonoses, Shandong Agricultural University, 7 Panhe Street, Tai’an 271017, China

**Keywords:** bovine mammary epithelial cell, *Escherichia coli*, *Lactobacillus plantarum* 17-5, inflammation, apoptosis, cGAS-STING, gut–mammary axis

## Abstract

*Escherichia coli* (*E. coli)* is a major etiological agent of clinical bovine mastitis. This study evaluated the therapeutic potential of *Lactobacillus plantarum* 17-5 (*LP*) against *E. coli*-induced mastitis via clinical, animal, and cellular models. In a trial with mastitic dairy cows, dietary *LP* significantly reduced systemic inflammatory markers (IL-6, IL-1β, TNF-α) by 2–3-fold (*p* < 0.05) and milk somatic cell count by 7-fold (*p* < 0.05). 16S rRNA sequencing revealed these improvements were associated with substantial gut microbiota restructuring, suggesting a link between gut microbial balance and mammary health via the gut–mammary axis. In a murine model, *LP* mitigated mammary inflammatory injury (histopathology) and restored tight junction integrity while reducing apoptosis (western blot, *p* < 0.05). In bovine mammary epithelial cells (BMECs), *LP* suppressed the cGAS-STING pathway, inhibiting NF-κB P65 phosphorylation and downstream pro-inflammatory cytokine production (*p* < 0.05). Collectively, *LP* alleviates *E. coli*-associated mastitis by modulating gut microbiota through the gut–mammary axis and directly inhibiting the cGAS-STING/NF-κB axis, supported by multi-model evidence.

## Simple Summary

Mastitis, often caused by *Escherichia coli*, is a widespread and costly problem for the dairy industry. This study explored whether a probiotic called *Lactobacillus plantarum 17-5* could serve as a natural alternative. We found that feeding this probiotic to cows with mastitis reduced inflammation and improved gut health, which in turn helped restore udder health. In further tests on mice and bovine mammary epithelial cells, we discovered that the probiotic protects the mammary tissue by strengthening its natural barrier and blocking a key inflammatory pathway known as cGAS-STING. These findings suggest that *Lactobacillus plantarum 17-5* could be a safe and effective supplement to help prevent or treat mastitis, reducing the need for antibiotics in dairy farming.

## 1. Introduction

Bovine mastitis is universally recognized as the most significant economic and animal welfare challenge in global dairy production [1]. *E. coli* is a major etiological agent of bovine mastitis [2]. *E. coli* induces inflammation ranging from mild subclinical cases to severe clinical mastitis characterized by toxemia, high fever, and reduced milk production [3,4]. And it employs virulence mechanisms such as fimbrial/pili-mediated adhesion and effector protein secretion to subvert host cell functions, disrupt immune responses, and establish persistent colonization [5].

Antibiotics are routinely used prophylactically against mastitis in dairy cows throughout the dry period [6]. Antibiotic overuse and treatment failures highlight the urgent need for effective strategies to reduce bacterial mastitis incidence.

Probiotics are often used as an alternative to antibiotics, with lactobacilli in particular exerting beneficial effects on the host and alleviating adverse side effects induced by antibiotics [7]. Gut probiotics affect distant organs by secreting metabolites that influence their functions [8]. Studies have shown that various bacteria capable of producing anti-inflammatory metabolites through metabolism can alleviate symptoms of bacterial mastitis [9]. *LP* is a commonly used lactobacillus feed additive and is widely utilized in animal husbandry. It exhibits potent antimicrobial activity against *E. coli* via bacteriocin production—ribosomally synthesized antimicrobial peptides that function as natural preservatives and antibiotic adjuvants [10]. Furthermore, *LP* enhances intestinal and mammary barrier integrity, modulates TLR-mediated inflammatory pathways [11], and restores redox balance by upregulating antioxidant enzymes. Research has shown that the metabolites of *LP*, SCFAs, help maintain the host’s health [12].

The cGAS-STING signaling pathway has emerged as a crucial mediator of innate immunity. The pathway is capable of recognizing pathogen DNA and its metabolites, and thereby contributes to a variety of inflammatory injury-related diseases [13]. Bovine mastitis is primarily triggered by pathogens such as *Escherichia coli*, and the connection between its pathogenesis and the cGAS-STING pathway remains to be explored. Although some studies demonstrated the various effects of LP [14], it remains unclear whether *LP* exerts its anti-mastitis effects through the cGAS-STING signaling pathway. Given that the cGAS-STING pathway recognizes pathogenic DNA and triggers inflammation, we hypothesized that LP could prevent mastitis in dairy cows by modulating the cellular cGAS-STING signaling pathway. Therefore, this study aimed to validate this hypothesis and investigate the underlying mechanism. Our research seeks to provide mechanistic insights into *LP*’s anti-mastitis effects to support the development of probiotic strategies as alternatives to antibiotics in dairy cattle

## 2. Materials and Methods

### 2.1. Experimental Animal Management and Group Assignment

All animal experiments were approved by the Animal Care and Use Committee of the College of Veterinary Medicine, Shandong Agricultural University (Protocol Number: SDAUA-2024-172).

For the bovine experiments, a total of fourteen Chinese Holstein dairy cows were selected. The selection criteria included: (1) age between 3 to 5 years; (2) similar parity of 2nd to 3rd lactation; (3) mid-lactation stage (60–150 days postpartum). All animals were provided by a large-scale dairy farm in Dongying City, Shandong Province, and were randomly allocated into experimental and control groups with balanced physiological conditions. Dairy cows were allocated into two groups of seven [15]: a healthy Control (Con, SCC ≤ 500,000/mL) and a mastitis Model (Mod, SCC > 500,000/mL, *E. coli* culture positive) group. The severity of clinical mastitis in the experimental cows was assessed according to the National Mastitis Council (NMC) criteria, with observed symptoms including redness and swelling of the udder, as well as the presence of flakes and clots in the milk. The Model group subsequently received oral *LP* (120 g/head/day) for 14 days and was redesignated as the Treatment (Tre) group. Blood and fecal samples were collected from all groups on days 0 and 14.

For mouse experiments, thirty-two lactating Kunming mice (7–10 days postpartum) were randomly assigned to four groups (*n* = 8 per group) [16]: CON (PBS), EC (*E. coli*, 1.0 × 10^7^ CFU/50 μL), three LP+EC groups (*LP* pre-treatment at 1.0 × 10^7^ CFU/50 μL for 3 h, followed by *E. coli*), and LP (*LP* alone). Under ether anesthesia, all groups received intramammary injections via the exposed teat canal. Mice were euthanized at 24 h post-infection for mammary tissue collection.

### 2.2. Bacteria and the Culture Conditions

The challenge experiments utilized two bacterial strains: (1) *Escherichia coli* CVCC1450 (serotype O111:K58), a bovine mastitis-derived pathogen kindly provided by the Clinical Veterinary Medicine Laboratory of Shandong Agricultural University; (2) the reference strain *Lactobacillus plantarum* ATCC 8014, purchased from the Shanghai Bio-Culture Collection Center (SHBCC).

For *E. coli* inoculation, a single colony was inoculated into LB broth (Hopebio, Qingdao, China) and cultured aerobically overnight at 37 °C. This pre-culture was then diluted 1:5 in fresh medium and incubated for an additional 12 h to reach the mid-logarithmic growth phase (OD_600_ = 0.5), yielding approximately 10^8^ CFU/mL.

For *LP*, a similar two-step cultivation was performed anaerobically in MRS broth (Hopebio, Qingdao, China) at 37 °C. The stationary phase (OD_600_ = 1.0, ~10^9^ CFU/mL) was achieved after 24 h of incubation.

Cells from both cultures were harvested by centrifugation (4000× *g*, 10 min), washed twice, and resuspended in sterile PBS.

### 2.3. Cell Culture and Treatment

BMECs were acquired by the Veterinary Clinical Laboratory of Shandong Agricultural University. The cells were maintained with DMEM-F12 medium (Servicebio, Wuhan, China) with 10% fetal bovine serum (FBS, TianHang Biotech, Huzhou City, China) and were used for experiments between passages 3 and 8. Cells were seeded into culture dishes and incubated at 37 °C under 5% CO_2_ until they reached the desired confluence.

For the experiments, cells at 70–80% confluence were assigned to the following groups: CON (medium only), EC (*E. coli*, 10^5^ CFU/mL, 5 h), LP (*LP*, 10^5^ CFU/mL, 3 h), and LP+EC (*LP* pre-treatment for 3 h followed by *E. coli* for 5 h). After *E. coli* exposure, the medium was discarded in all groups. C-176 (Selleck, Houston, TX, USA) was first dissolved in DMSO to prepare a 15 mM stock solution. For the experiments, all relevant cell groups were pre-treated with DMEM-F12 medium containing 20 μM C-176 (diluted from the stock solution) for 12 h.

### 2.4. Colorimetric and ELISA Assays

The following assays were performed using commercial kits according to the manufacturers’ instructions: myeloperoxidase (MPO) activity (Jiancheng Bioengineering Institute, Nanjing, China) and cytokine ELISA (Jingma, Shanghai, China). All ELISA samples were assayed in duplicate.

### 2.5. Microbial DNA Extraction and 16S rRNA Sequencing

Fecal samples were collected for bacterial DNA extraction. The V3-V4 region of the 16S rRNA gene was amplified using PCR with the following reaction system: 15 μL of Phusion^®^ High-Fidelity PCR Master Mix, 0.2 μM of each primer (515F-806R, specific for 16S rRNA gene V4 region), and approximately 10 ng of template DNA. The thermal cycling protocol included an initial denaturation at 98 °C for 1 min, followed by 30 cycles of denaturation (98 °C, 10 s), annealing (50 °C, 30 s), and elongation (72 °C, 30 s), with a final extension at 72 °C for 5 min. The PCR products were quantified and qualified via electrophoresis on a 2% agarose gel, followed by magnetic bead purification (detailed quality control criteria refer to the accompanying QC report). Subsequently, equimolar amounts of the purified products were pooled for library construction. Sequencing libraries were generated with index addition, quantified using Qubit and real-time PCR, and assessed for size distribution via a bioanalyzer. The pooled libraries were then sequenced on an Illumina NovaSeq 6000 platform (consistent with alternative optional platform DNBSEQ-G99) according to the required data output. The raw sequencing data generated in this study will be deposited in the NCBI Sequence Read Archive (SRA) database, and the accession number will be provided upon manuscript acceptance.

Statistical and Bioinformatics Analysis:

All bioinformatics analyses were conducted using QIIME2 and R software (Version 4.0.3). Briefly: (1) Paired-end reads were assigned to samples by unique barcodes (barcode/primer sequences truncated), merged with FLASH (V1.2.11), filtered with fastp (V0.23.1) to obtain clean tags, and chimera-removed with vsearch (V2.16.0) against the Silva database (16S rRNA gene) to get effective tags; (2) ASV (Amplicon Sequence Variants) denoising was performed via the DADA2 module in QIIME2, and species annotation used the Silva database (taxonomic information supplemented with NCBI dmp file for unannotated sequences); (3) Alpha diversity (Observed_otus, Chao1, Shannon, Simpson, Dominance, Good’s coverage, Pielou_e) was calculated in QIIME2, with species accumulation boxplots generated via the vegan package (available in R v4.0.3); (4) Beta diversity was analyzed based on weighted/unweighted Unifrac distances (QIIME2), with visualization via beta diversity heatmaps (Perl), UPGMA cluster trees (QIIME2), PCA/PCoA/NMDS (R v4.0.3 ade4 and ggplot2 packages); (5) Community difference analysis included Anosim/Adonis/MRPP/Simper (vegan package), MetagenomeSeq (metagenomeSeq package), and LEfSe (v1.1.01); (6) Functional prediction was conducted with PICRUSt2 (V2.3.0) and Tax4Fun (V0.3.1).

### 2.6. Cell Counting Kit-8 Assay

Inoculate BMECs into 96-well plates and wait for the cells to grow to 70–80%. Cell viability was assessed using Cell Counting Kit-8 (CCK-8; NCMbio, Suzhou, China).

### 2.7. Western Blot

Cell pellets underwent three 1 mL washes with ice-cold PBS, followed by 20-min on-ice lysis in buffer (G-Clone, Beijing, China). For denaturation, proteins were mixed with 5 ×loading buffer and heated at 100 °C in a metal bath for 4 min. The SDS-PAGE technique was applied to achieve separation by molecular weight. The separated proteins were subsequently immobilized on polyvinylidene fluoride (PVDF) membranes. Membranes were blocked (5% BSA/TBST, 1 h), incubated with primary antibodies (4 °C, overnight), washed (3 × 10 min TBST), then secondary antibodies (1:10,000, 1 h). Visualized protein bands were detected using ECL (NCMbio, Suzhou, China) reagent, and band density was assessed using imaging software (ImageJ 1.53e). Antibody specifications are provided in Supplementary Table S1.

### 2.8. Real-Time Quantitative PCR1

Total RNA extraction from BMECs was performed using TRIzol reagent (NCMbio, Suzhou, China) following standard protocols. cDNA synthesis and RT-qPCR were performed with a commercial kit (Accurate Biology, Changsha City, China) according to the manufacturer’s protocol. The thermal cycling protocol consisted of: Initial denaturation at 95 °C for 2 min; 40 amplification cycles (95 °C 20 s, 60 °C 30 s). Gene expression was normalized to GAPDH and analyzed via the 2^−ΔΔCt^ method (primer sequences in Table S2).

### 2.9. Annexin V-FITC/PI Staining

Following treatment, cells were harvested and analyzed for apoptosis using Annexin V-FITC/PI staining (Vazyme, Nanjing, China) according to the manufacturer’s protocol. Flow cytometry was performed, and apoptosis rates were quantified with FlowJo software (FlowJo 10.8.1).

### 2.10. Immunofluorescence

BMECs cultured on coverslips in 24-well plates (70–80% confluency, optimal for immunofluorescence) were exposed to bacterial suspensions. Cells on coverslips were fixed with 4% paraformaldehyde (15 min, RT), blocked with 5% BSA (1 h), incubated with primary antibodies (4 °C overnight) and fluorescent secondary antibodies (1 h, dark), then mounted with DAPI-containing medium (NCMbio, Suzhou, China). Confocal imaging was performed. Follow the manual instructions for Hoechst/PI staining.

### 2.11. Hematoxylin and Eosin Staining

Following deparaffinization and hydration through a graded series of xylene and ethanol, paraffin-embedded sections were washed in running water. Frozen sections were thawed to room temperature and fixed. All sections were then subjected to antigen retrieval, stained with hematoxylin and eosin (H&E), dehydrated, cleared in xylene, and finally mounted with neutral balsam for microscopic examination. Mammary gland histology was evaluated using a scoring system that assessed both acinar structure destruction (0: none; 1: slight; 2: moderate; 3: severe) and inflammatory cell infiltration (0: none; 1: slight; 2: moderate; 3: severe) [17].

### 2.12. Statistical Analysis

Data are presented as mean ± standard deviation (mean ± SD) from at least three independent biological replicates. All statistical analyses were conducted using SPSS software (version 26.0). Depending on the experimental design, either one-way or two-way analysis of variance (ANOVA) was employed. For multiple group comparisons involving a single factor, one-way ANOVA was applied, followed by the LSD post hoc test for pairwise comparisons. For factorial designs (*LP* and *E. coli* interaction), two-way ANOVA was used to assess main effects and their interactions. In all cases, a *p*-value < 0.05 was considered statistically significant.

## 3. Results

### 3.1. LP Significantly Alleviates Mastitis and Modulates the Gut Microbiota in Dairy Cows

**Figure 1:**
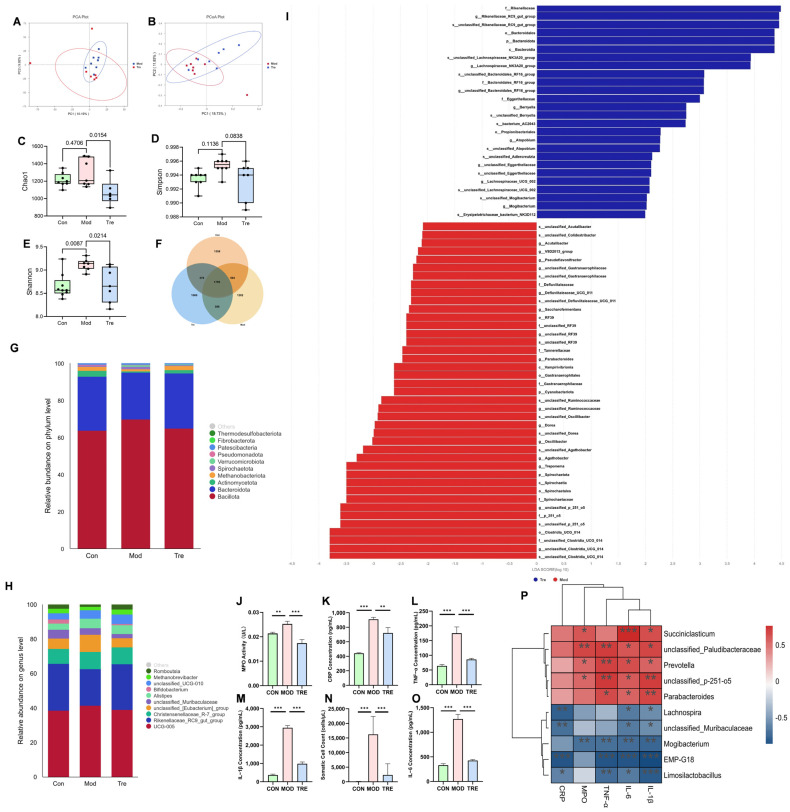
Effects of *LP* supplementation on gut microbiota and systemic inflammation in dairy cows. (**A**,**B**) PCoA of fecal microbiota. (**C**–**E**) Alpha diversity indices (Observed, Chao1, Shannon). (**F**) Venn diagram of OTUs. (**G**) Relative abundance of the top 10 phyla. (**H**) Relative abundance of the top 10 genera. (**I**) LEfSe analysis identifying differentially enriched taxa between Mod and Tre groups. (**J**–**O**) Milk somatic cell count (SCC) and serum levels of inflammatory markers (IL-6, IL-1β, TNF-α, MPO, CRP). (**P**) Spearman correlation analysis between significant bacterial genera and inflammatory parameters. Red and blue circles indicate positive and negative correlations, respectively, with color intensity representing the strength of the correlation. Data are presented as the mean ± SD from at least three independent experiments (*n* ≥ 3). Statistical analyses were performed using one-way or two-way ANOVA, as appropriate, followed by the LSD post hoc test for specific pairwise comparisons. A *p*-value of less than 0.05 was considered statistically significant (* *p* < 0.05, ** *p* < 0.01, *** *p* < 0.001).

We collected a total of 21 fecal and serum samples from 7 dairy cows with clinical mastitis and 7 matched healthy cows before and after the trial, based on clinical diagnosis and blood cell count. Principal coordinate analysis (PCoA) score plots of the fecal samples revealed a clear separation between the Mod and Tre groups (Figure 1A,B). The microbial diversity and richness in the Mod group were significantly higher than those in the Con and Tre groups, and these indices decreased after supplementation with *LP* (Figure 1C–E). Venn analysis of the gut microbial community showed differences in the number of operational taxonomic units (OTUs) among the Con, Mod, and Tre groups (Figure 1F). Significant differences in gut microbial composition were observed among these three groups at both the phylum and genus levels (Figure 1G, H). Additionally, LEfSe analysis identified distinct shifts in the microbial community between the Mod and Tre groups. The analysis showed that 15 genera were associated with the mastitis condition (Mod), while a contrasting set of 8 genera was enriched after probiotic intervention (Tre, Figure 1I). The results of serum MPO and inflammatory markers showed that inflammatory marker levels were significantly higher in the Mod group than in the Con group, and decreased significantly after probiotic supplementation (Figure 1J–O). Furthermore, Spearman correlation analysis between different bacterial genera and inflammatory parameters revealed that 5 genera, including Succiniclasticum, were positively correlated with inflammatory factors, while 5 other genera, including Lachnospira, showed a negative correlation with inflammatory marker levels (Figure 1P).

### 3.2. LP Significantly Alleviated Inflammatory Injury in the Mammary Gland of Mice

**Figure 2:**
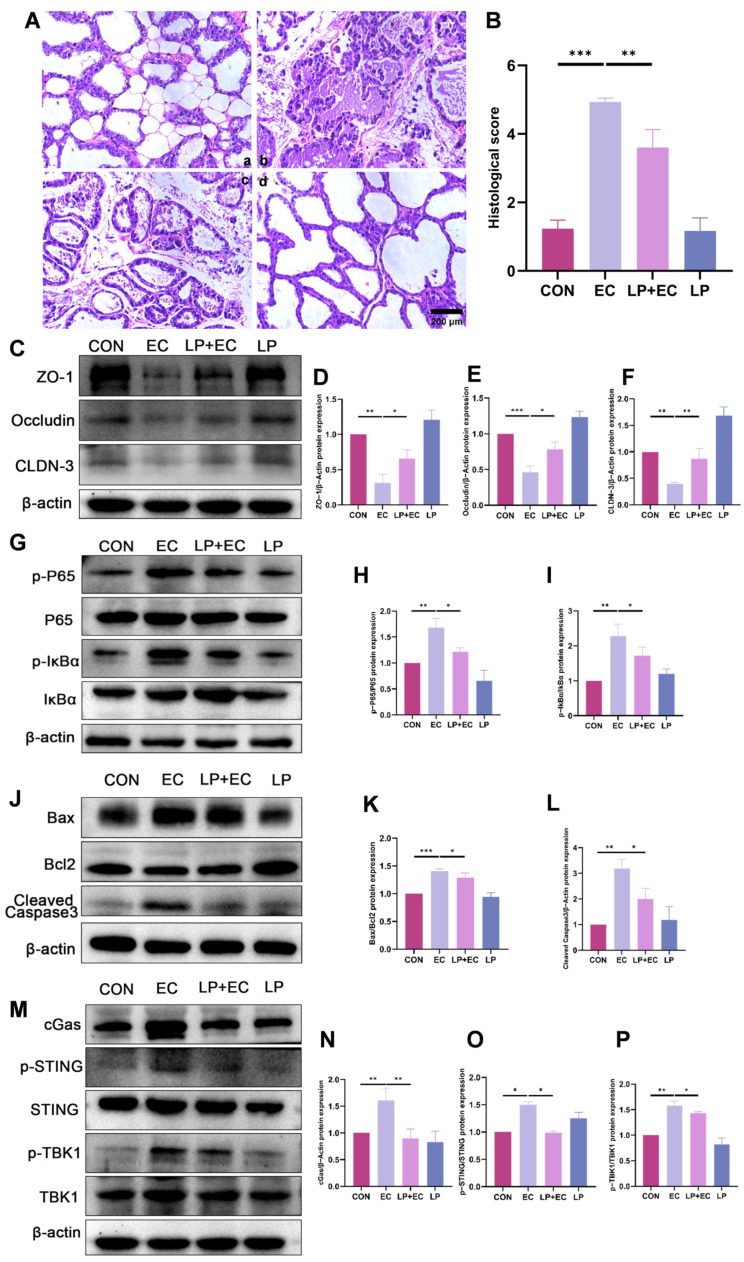
*LP* alleviates *E. coli*-induced mastitis in mice by protecting mammary tissue integrity and suppressing inflammatory signaling. (**A**,**B**) Histopathological analysis of murine mammary tissue. (**A**) Representative H&E-stained tissue sections from different treatment groups: (**a**) Control, (**b**) *E. coli* -infected, (**c**) *LP* (1.0 × 10^7^ CFU/100 μL) pretreatment followed by *E. coli* challenge, (**d**) *LP* alone. Scale bar = 200 µm. (**B**) Quantitative histological injury scores for each group. A higher score indicates more severe damage. (**C**–**F**) WB analysis of TJ protein expression. Protein levels of ZO-1, Occludin, and Claudin-3 (Cldn-3) in murine mammary tissue were quantified by WB following *LP* treatment. (**G**–**I**) WB analysis of P65 and IκBα phosphorylation. Phosphorylation levels of P65 and IκBα proteins were assessed by WB in murine mammary tissue. (**J**–**L**) WB results for protein expression of Cleaved Caspase3, Bax, and Bcl2. (**M**–**P**) WB analysis of cGAS-STING pathway protein expression. Data are presented as the mean ± SD from at least three independent experiments (*n* ≥ 3). Statistical analyses were performed using one-way or two-way ANOVA, as appropriate, followed by the LSD post hoc test for specific pairwise comparisons. A *p*-value of less than 0.05 was considered statistically significant (* *p* < 0.05, ** *p* < 0.01, *** *p* < 0.001).

To investigate the therapeutic effect of *LP* on mastitis, hematoxylin and eosin (H&E) staining was performed on mammary gland tissues from the different experimental mouse groups. The results revealed that mammary gland injection with *E. coli* significantly disrupted mammary tissue architecture. *LP* pretreatment reduced injury scores by 73% (Figure 2A,B). To explore the molecular mechanisms underlying this protection, we performed western blot (WB) analysis. The results demonstrated that *LP* pretreatment effect- tively mitigated mastitis-induced injury, as evidenced by the restoration of tight junction (TJ) proteins (Figure 2C–F), suppression of inflammatory responses (Figure 2G–I), and reduced apoptosis levels (Figure 2J–L). Furthermore, *E. coli* challenge significantly upregulated the expression of proteins associated with the cGAS-STING signaling pathway in the mammary gland. This upregulation was notably suppressed in mice that received oral administration of *LP* prior to induction (Figure 2M–P).

### 3.3. LP Alleviates E. coli-Induced Apoptosis and Inflammatory Response in BMECs

**Figure 3:**
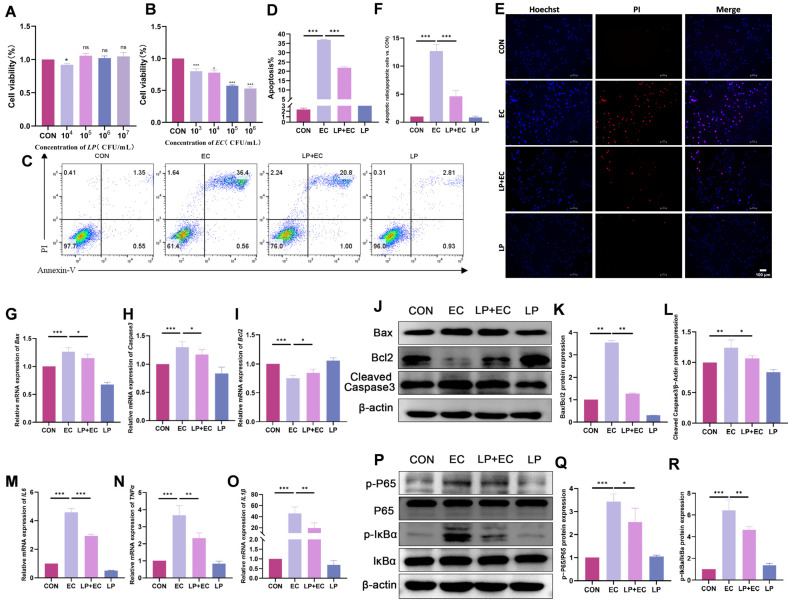
*LP* alleviates *E. coli*-induced apoptosis and inflammatory response in BMECs. (**A**) Effect of *LP* on BMEC viability. Cells were treated with the indicated concentrations of *LP* for 3 h. (**B**) Effect of *E. coli* on BMEC viability. Cells were treated with the indicated concentrations of *E. coli* for 5 h. Cell viability was determined by CCK-8 assay and expressed as a percentage of the control group (0 CFU/mL). (**C**,**D**) Flow cytometry analysis of apoptosis. BMECs were pretreated with or without *LP* (10^6^ CFU/mL) for 3 h followed by challenge with *E. coli* (10^5^ CFU/mL) for 5 h. Representative flow cytometry plots (**C**) and quantitative analysis of apoptosis rates (**D**) are shown. (**E**,**F**) Fluorescence staining for apoptosis and necrosis assessment. After treatments, cells were stained with Hoechst 33,342 (blue, nuclei) and propidium iodide (PI, red, necrotic cells). Apoptotic cells are characterized by condensed and bright blue nuclei, while necrotic cells show red PI staining. Scale bar = 100 µm (applicable to all panels in (**E**)). A representative image from each group is shown (**E**), with quantitative analysis of apoptotic and necrotic cells presented in (**F**). (**G**–**I**) mRNA expression levels of apoptosis-related genes (*Caspase3*, *Bax*, *Bcl2*) determined by RT-qPCR. (**J**–**L**) Protein expression levels of Cleaved Caspase3, Bax, and Bcl2 determined by WB. β-actin was used as a loading control. (**M**–**O**) mRNA expression levels of inflammatory cytokines *(IL-1β*, *IL-6*, *TNF-ạ*) determined by RT-qPCR. (**P**–**R**) Protein expression levels of p-P65, total P65, p-IκBα, and total IκBα determined by WB. Data are presented as the mean ± SD from at least three independent experiments (*n* ≥ 3). Statistical analyses were performed using one-way or two-way ANOVA, as appropriate, followed by the LSD post hoc test for specific pairwise comparisons. A *p*-value of less than 0.05 was considered statistically significant (* *p* < 0.05, ** *p* < 0.01, *** *p* < 0.001).

To elucidate the protective mechanism of *LP* against mastitis, we first examined its effect on apoptosis in BMECs. The CCK-8 assay showed that while *LP* alone (10^5^–10^7^ CFU/mL) had no significant effect on cell viability, *E. coli* challenge (10^3^–10^6^ CFU/mL, 5 h) markedly reduced it. Consequently, a condition of 10^5^ CFU/mL *E. coli* for 5 h was selected for subsequent experiments (Figure 3A,B). *LP* preserved BMEC viability and significantly reduced *E. coli*-induced apoptosis and necrosis, as confirmed by Annexin V/PI staining combined with fluorescence microscopy (Figure 3C–F). Consistently, RT-qPCR analysis revealed that *LP* pretreatment restored the expression of apoptosis-related genes, decreasing the pro-apoptotic Bax to 91% (*p* = 0.032) and increasing the anti-apoptotic Bcl2 to 113% (*p* = 0.045) of the model level, thereby reversing the Bax/Bcl2 ratio. Furthermore, the expression of the apoptosis executor Caspase3 was downregulated to 90% (*p* = 0.038) by *LP* treatment (Figure 3G–I). In support of the anti-apoptotic findings, WB results also confirmed a marked reduction in Bax protein expression to 0.36-fold (*p* < 0.001) and cleaved Caspase3 to 0.87-fold (*p* = 0.02) (Figure 3J–L).

We next examined whether *LP* alleviates the *E. coli*-triggered inflammatory response. RT-qPCR analysis indicated that *LP* significantly inhibited the upregulation of key inflammatory cytokines at the mRNA level, reducing IL-1β to 0.42-fold (*p* = 0.003), IL-6 to 0.63-fold (*p* < 0.001), and TNF-α to 0.62-fold (*p* < 0.001) compared to the *E. coli*-challenged group (Figure 3M–O). Correspondingly, WB analysis showed that *LP* suppressed the *E. coli*-induced activation of the NF-κB pathway. The phosphorylation level of P65 (p-P65/P65) was reduced to 0.74-fold (*p* = 0.014), and the phosphorylation level of IκBα (p-IκBα/IκBα) was decreased to 0.72-fold (*p* = 0.002) upon *LP* treatment (Figure 3P–R). These results collectively demonstrate that *LP* mitigates *E. coli*-induced inflammatory and apoptotic injury in BMECs by modulating the NF-κB signaling pathway.

### 3.4. LP Mitigates E. coli-Induced Damage to Tight Junctions

**Figure 4:**
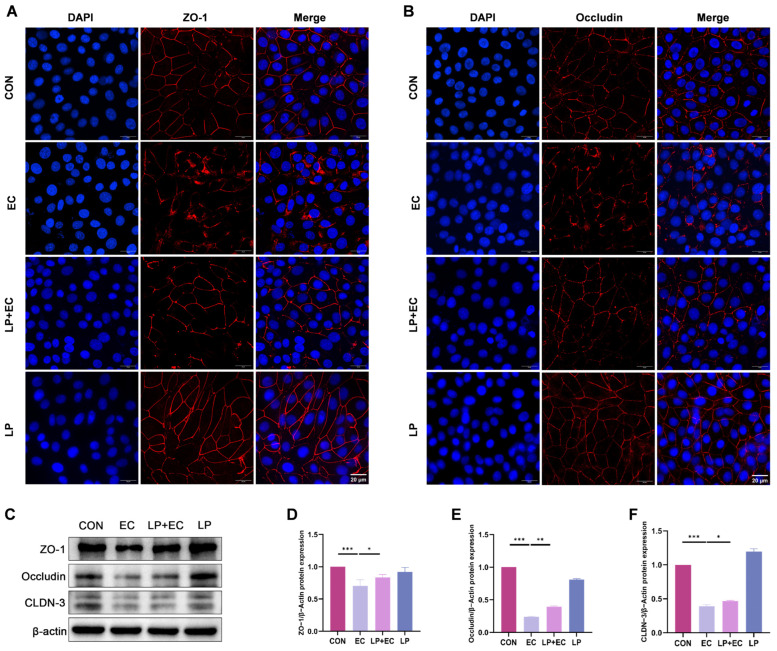
Effects of *LP* on TJ integrity in BMECs. (**A**,**B**) Immunofluorescence imaging of TJ proteins in BMECs. Fluorescence signals for Occludin and ZO-1 (red fluorescence) and nuclear staining with DAPI (blue fluorescence) are shown. BMECs were treated with *LP* to assess changes in TJ protein localization. Scale bar = 20 μm (applicable to all panels in (**A**,**B**)) (**C**–**F**) WB analysis of TJ protein expression. Protein levels of ZO-1, Occludin, and CLDN-3 in BMECs were quantified by WB following *LP* treatment. Data are presented as the mean ± SD from at least three independent experiments (*n* ≥ 3). Statistical analyses were performed using one-way or two-way ANOVA, as appropriate, followed by the LSD post hoc test for specific pairwise comparisons. A *p*-value of less than 0.05 was considered statistically significant (* *p* < 0.05, ** *p* < 0.01, *** *p* < 0.001).

To investigate the effect of LP on TJ integrity, we analyzed the expression and lo-calization of key TJ proteins (ZO-1, Occludin, and CLDN-3) in BMECs. Immunofluo-rescence staining revealed that in the control group, these proteins exhibited a continuous and sharply defined linear distribution at the cell boundaries, whereas *E. coli* challenge severely disrupted their organization, leading to fragmentation and diminished fluorescence signal. At the protein level, WB analysis consistently showed a significant downregulation in the expression of ZO-1, Occludin, and CLDN-3 following *E. coli* infection (*p* < 0.05). Importantly, *LP* pretreatment markedly attenuated these alterations: it not only restored the membrane-localized expression and continuous distribution of TJ proteins as visualized by fluorescence microscopy (*p* < 0.05, Figure 4A,B)., but also significantly upregulated their protein expression compared to the *E. coli*-treated group (*p* < 0.05, Figure 4C–F). These results collectively demonstrate that *LP* directly enhances the expression and promotes the proper organization of TJ proteins, thereby contributing to the restoration of blood-milk barrier integrity.

### 3.5. LP Exerts a Protective Effect by Inhibiting the cGAS-STING Pathway

**Figure 5:**
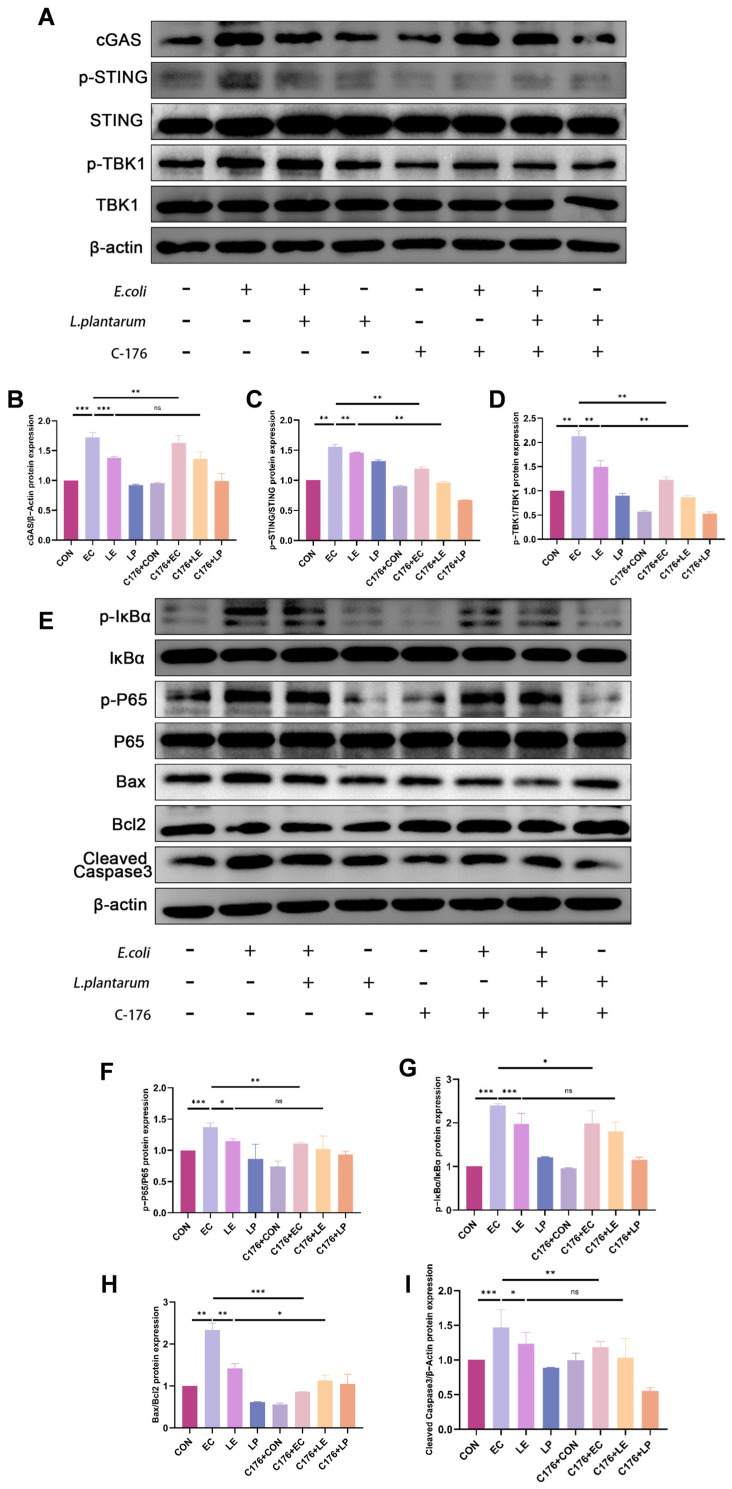
*LP* alleviates *E. coli*-induced injury in BMECs by modulating the cGAS-STING signaling pathway. (**A**–**D**) WB analysis of cGAS-STING pathway protein expression with or without STING inhibition. BMECs were treated with *LP* and/or the STING inhibitor C-176. Protein levels of cGAS, phosphorylation level of STING and TBK1 were assessed by WB to evaluate *LP*’s regulatory effects on the cGAS-STING pathway. (**E**–**I**) WB analysis of inflammatory and apoptotic protein expression following STING inhibition. The symbols "+" and "−" indicate the presence and absence of the treatment, respectively. Data are presented as the mean ± SD from at least three independent experiments (*n* ≥ 3). Statistical analyses were performed using one-way or two-way ANOVA, as appropriate, followed by the LSD post hoc test for specific pairwise comparisons. A *p*-value of less than 0.05 was considered statistically significant (* *p* < 0.05, ** *p* < 0.01, *** *p* < 0.001).

To explore the mechanism by which *LP* mitigates mastitis, we examined its regulatory effects on the cGAS-STING signaling pathway. WB analysis demonstrated that *E. coli* infection markedly upregulated the expression of cGAS and Bax/Bcl2, along with the cleavage of caspase 3 and phosphorylation levels of STING, TBK1, IκBα, and P65. In contrast, *LP* treatment significantly suppressed these pathophysiological alterations induced by *E. coli* infection. To further validate the pathway-specific role, cells were treated with the STING inhibitor C-176. The results showed that C-176 significantly inhibited *E. coli*-induced activation of the cGAS-STING pathway and associated inflammatory injury; however, it did not markedly influence the protective effects of *LP* (*p* < 0.05, Figure 5A–I). In general, these findings suggest that *LP* alleviates damage to BMECs by modulating the cGAS-STING signaling pathway.

## 4. Discussion

The commensal microbiota residing within the healthy mammary gland are pivotal in sustaining immune homeostasis [18]. Conversely, studies have established that gut microbiota dysbiosis enables bacterial translocation via the entero-mammary pathway through lymphatic and hematogenous routes, ultimately inducing bovine mastitis [19,20]. Given this crucial role of microbial balance, we sought to investigate the alleviative effect of the probiotic *LP* on dairy cow mastitis. We administered the probiotic to both healthy and mastitis-affected cows and measured relevant indicators. The results demonstrated that clinical mastitis is associated with a systemic inflammatory state and a profound restructuring of the gut microbial community. This was confirmed by principal coordinate analysis, which revealed a clear separation in gut microbial structure between mastitic (Mod) and healthy (Con) cows. Specifically, the Mod group exhibited higher microbial diversity and richness, a pattern often indicative of a dysbiotic state characterized by community imbalance rather than a simple loss of diversity, as supported by the enrichment of genera like Treponema. Supplementation with *LP* effectively mitigated these changes. The Tre group showed a reduction in diversity and richness towards healthy levels, coupled with a significant enrichment of beneficial genera such as Lachnospira, a known producer of anti-inflammatory short-chain fatty acids [21]. This microbial remodeling was accompanied by a significant decrease in systemic inflammatory markers, suggesting that *LP* exerts its anti-inflammatory effects through gut microbiota modulation. Correlation analysis further strengthened the link between specific gut bacteria and systemic inflammation. The positive correlation of genera like Succiniclasticum with inflammatory factors, and the negative correlation of genera like Lachnospira, provides compelling evidence for the involvement of the gut–mammary axis in mastitis. These findings were further supported by in vivo experiments in mice, where pre-administration of *LP* alleviated inflammatory cell infiltration in mammary tissue and reduced the expression of inflammatory injury-related proteins in mastitis model mice compared to the inflammation model group. Notably, we observed a downregulation in the expression of proteins associated with the cGAS-STING signaling pathway in mouse mammary tissue following oral administration of *LP*. The ability of *LP* to modulate gut microbiota through dynamic interactions with commensal microorganisms underscores its potential as a strategic biotherapeutic agent for combating antimicrobial-resistant infections in veterinary medicine [22,23].

In BMECs, the blood-milk barrier integrity—maintained by tight junction (TJ) proteins such as ZO-1, Occludin, and CLDN-3—is critical for normal mammary physiology [24]. This barrier is disrupted during mastitis, as pathogens like *E. coli* induce cytokine-driven degradation and direct damage to TJ proteins [25]. Pathogen-induced mastitis disrupts TJ dynamics [26] via cytokine-driven protein degradation and direct junctional damage, with *E. coli* causing severe TJ disruption [27,28]. Our experiments demonstrated that *LP* treatment effectively restored the expression and distribution of these TJ proteins in *E. coli*-challenged BMECs, thereby counteracting the barrier dysfunction central to mastitis pathophysiology.

Mastitis is typically accompanied by inflammatory and apoptotic processes [29]. The intracellular contents released by apoptotic cells trigger an inflammatory response [30]. To investigate the changes in inflammation and apoptosis, we investigated the concomitant inflammatory and apoptotic processes in mastitis. Our results showed that *E. coli* infection triggered both a strong inflammatory response, via NF-κB pathway activation and pro-inflammatory cytokine (TNF-α, IL-6, IL-1β) release, and apoptotic cell death. *LP* treatment effectively mitigated these responses. It exerted anti-inflammatory effects by suppressing the NF-κB pathway and reducing cytokines. Furthermore, *LP* attenuated apoptosis by modulating the mitochondrial Bax/Bcl-2 balance, a finding corroborated by the observation of reduced cellular shrinkage and a lower apoptotic rate via Annexin V staining [31,32]. This surface alteration is mediated by calcium-dependent phospholipid scrambling [33]. By preserving mitochondrial integrity and regulating caspase-3 activation, *LP* maintained controlled cell deletion essential for tissue homeostasis [34], demonstrating its dual role in inflammation and apoptosis mitigation. In this study, both inflammation and apoptosis in *E. coli*-induced BMECs were alleviated following *LP* treatment, indicating that *LP* can protect BMECs from damage by mitigating inflammatory responses and apoptosis.

**Figure 6:**
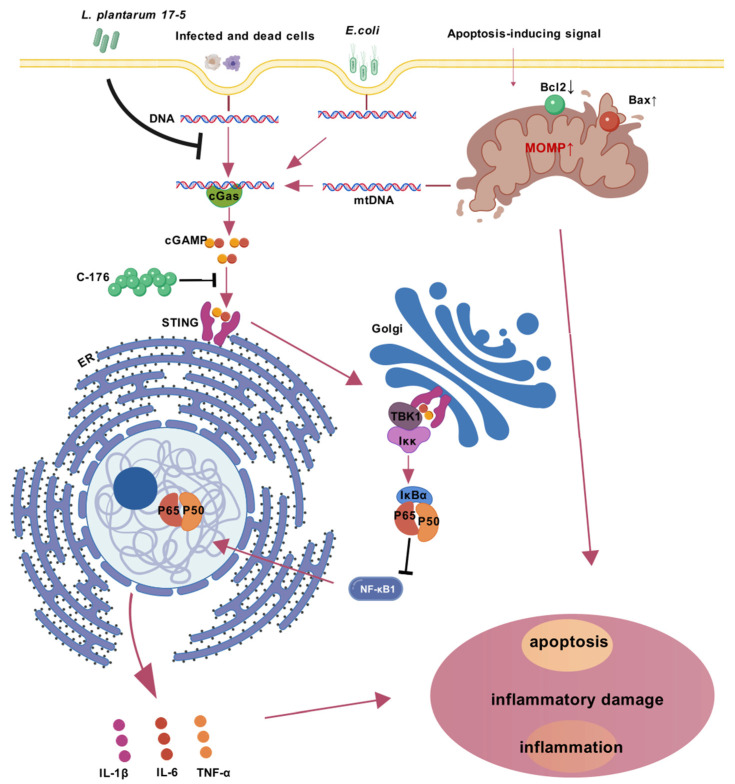
Mechanistic model of *E. coli*-induced cGAS-STING-NF-κB inflammatory axis activation and its suppression by *Lactobacillus plantarum 17-5* in BMECs. Upon *E. coli* invasion, bacterial DNA and host genomic DNA from infected cells accumulate in the cytoplasm, activating cyclic GMP-AMP synthase (cGAS). Concurrently, pro-apoptotic Bax activation and anti-apoptotic Bcl2 suppression induce mitochondrial outer membrane permeabilization (MOMP), caspase activation, and mitochondrial DNA (mtDNA) release, which synergistically amplify cGAS stimulation. cGAS synthesizes 2′3′-cGAMP to activate STING oligomerization (a process inhibited by C-176) on the endoplasmic reticulum (ER), triggering STING translocation to the Golgi apparatus. This facilitates TBK1 phosphorylation and subsequent IKK complex activation, leading to IκBα degradation and NF-κB nuclear translocation. The resultant transcription of proinflammatory cytokines (e.g., IL-6, TNF-α, IL-1β) exacerbates cellular inflammatory injury and apoptosis. Arrows signify the proposed sequence of causal events in the underlying mechanism. Intervention with *LP* attenuates this cascade by blocking STING oligomerization, suppressing TBK1/NF-κB signaling, and restoring mitochondrial apoptotic balance, thereby mitigating *E. coli*-induced inflammatory injury in BMECs.

The cGAS-STING signaling pathway serves as a cytoplasmic DNA surveillance system [35]. cGAS activation catalyzes 2′3′-cGAMP synthesis, which subsequently induces STING phosphorylation. This sequential activation of the signaling pathway ultimately initiates the transcriptional upregulation of NF-κB and type I interferon (IFN) genes [36]. The cGAS-STING signaling pathway plays a pivotal role in innate immunity by serving as a cytosolic DNA surveillance system that detects pathogenic invasions and mitochondrial DNA (mtDNA) leakage [37]. During pathogen challenge, compromised mitochondrial membrane integrity facilitates mtDNA release through two sequential mechanisms: permeabilization of the inner mitochondrial membrane (IMM) via MPTP formation [38], followed by outer mitochondrial membrane (OMM) rupture mediated by BAX/BAK macropores [39]. This liberated mtDNA subsequently activates the cGAS-STING cascade, triggering downstream inflammatory responses (Figure 6). At the same time, apoptotic caspases concurrently cleave cGAS/IRF3 to prevent inflammatory overactivation [40]. Our investigation revealed that *LP* exerts its protective effects through strategic modulation of this critical pathway. To functionally validate the involvement of STING, we employed an inhibitor that prevents activation-dependent palmitoylation of STING. Our experimental results revealed significant downregulation of both pro-inflammatory mediators and apoptosis-executing proteins upon STING inhibition.

Our work advances beyond previous descriptive studies by delineating a complete gut–mammary-immune pathway through which a probiotic exerts anti-inflammatory effects in mastitis. While earlier reports indicated that *LP* can modulate immunity, the explicit linkage to cGAS-STING suppression in the context of mammary infection represents a novel mechanistic insight. These results highlight the translational potential of *LP* as a probiotic-based strategy for mastitis management, providing a sustainable antibiotic alternative aligned with One Health objectives. However, critical challenges such as dosage scalability in commercial dairy herds, viability under field conditions, and long-term treatment efficacy remain to be addressed. Future studies should focus on practical formulation and large-scale trials to bridge the gap between mechanistic discovery and clinical implementation.

## 5. Conclusions

In conclusion, this study reveals *LP* as a multifaceted protector against *E. coli*-induced mammary damage, providing key mechanistic insight into its role in rebalancing the gut microbiota and mitigating systemic inflammation. These effects are predominantly mediated through suppression of the cGAS-STING pathway, underscoring the central role of the gut–mammary axis. Our findings establish a solid mechanistic foundation for the development of *LP*-based probiotics for veterinary use, highlighting their translational potential as a sustainable therapeutic alternative to conventional antibiotics in mastitis management.

## Data Availability

Data will be made available under reasonable requirements.

## Supplementary Materials

The following supporting information can be downloaded at: https://www.mdpi.com/article/10.3390/ani15223305/s1, Table S1: Antibody Information; Table S2: Sequences of primer used for RT-qPCR.

## References

[1] Sinha M.K., Thombare N.N., Mondal B. (2014). Subclinical mastitis in dairy animals: Incidence, economics, and predisposing factors. Sci. World J..

[2] Olson M.A., Cullimore C., Hutchison W.D., Grimsrud A., Nobrega D., De Buck J., Barkema H.W., Wilson E., Pickett B.E., Erickson D.L. (2024). Genes associated with fitness and disease severity in the pan-genome of mastitis-associated Escherichia coli. Front. Microbiol..

[3] Kumar P., Yang Z., Fatima H., Mitchell T. (2024). Hydroxyproline increases inflammation and Uropathogenic *E. coli* (UPEC) infection in female rats. Sci. Rep..

[4] Vangroenweghe F., Duchateau L., Burvenich C. (2020). Short communication: J-5 Escherichia coli vaccination does not influence severity of an Escherichia coli intramammary challenge in primiparous cows. J. Dairy Sci..

[5] Croxen M.A., Finlay B.B. (2010). Molecular mechanisms of Escherichia coli pathogenicity. Nat. Rev. Microbiol..

[6] Sharun K., Dhama K., Tiwari R., Gugjoo M.B., Iqbal Yatoo M., Patel S.K., Pathak M., Karthik K., Khurana S.K., Singh R. (2021). Advances in therapeutic and managemental approaches of bovine mastitis: A comprehensive review. Vet. Q..

[7] Hodzhev V., Dzhambazov K., Sapundziev N., Encheva M., Todorov S., Youroukova V., Benchev R., Nikolov R., Bogov B., Momekov G. (2024). High-dose Probiotic Mix of *Lactobacillus* spp., *Bifidobacterium* spp., *Bacillus coagulans*, and *Saccharomyces boulardii* to Prevent Antibiotic-associated Diarrhea in Adults: A Multicenter, Randomized, Double-blind, Placebo-controlled Trial (SPAADA). Open Forum Infect. Dis..

[8] Zhang Q., Chen D., Ding H., Li Q., Yuan S., Li H., Guan W., Zhang S. (2025). Lactobacillus amylovorus extracellular vesicles mitigate mammary gland ferroptosis via the gut–mammary gland axis. NPJ Biofilms Microbiomes.

[9] Li K., Ran X., Han J., Ding H., Wang X., Li Y., Guo W., Li X., Guo W., Fu S. (2025). Astragalus polysaccharide alleviates mastitis disrupted by Staphylococcus aureus infection by regulating gut microbiota and SCFAs metabolism. Int. J. Biol. Macromol..

[10] Yilmaz B., Bangar S.P., Echegaray N., Suri S., Tomasevic I., Manuel Lorenzo J., Melekoglu E., Rocha J.M., Ozogul F. (2022). The Impacts of Lactiplantibacillus plantarum on the Functional Properties of Fermented Foods: A Review of Current Knowledge. Microorganisms.

[11] Ren C., Zhang Q., de Haan B.J., Zhang H., Faas M.M., de Vos P. (2016). Identification of TLR2/TLR6 signalling lactic acid bacteria for supporting immune regulation. Sci. Rep..

[12] Mazziotta C., Tognon M., Martini F., Torreggiani E., Rotondo J.C. (2023). Probiotics Mechanism of Action on Immune Cells and Beneficial Effects on Human Health. Cells.

[13] Liu H., Hu Q., Ren K., Wu P., Wang Y., Lv C. (2023). ALDH2 mitigates LPS-induced cardiac dysfunction, inflammation, and apoptosis through the cGAS/STING pathway. Mol. Med..

[14] Hsu Y.C., Huang Y.Y., Tsai S.Y., Kuo Y.W., Lin J.H., Ho H.H., Chen J.F., Hsia K.C., Sun Y. (2023). Efficacy of Probiotic Supplements on Brain-Derived Neurotrophic Factor, Inflammatory Biomarkers, Oxidative Stress and Cognitive Function in Patients with Alzheimer’s Dementia: A 12-Week Randomized, Double-Blind Active-Controlled Study. Nutrients.

[15] Wang J., Li M., Wu W., Zhang H., Yang Y., Usman M., Aernouts B., Loor J.J., Xu C. (2024). Inflammatory Signaling via PEIZO1 Engages and Enhances the LPS-Mediated Apoptosis during Clinical Mastitis. J. Agric. Food Chem..

[16] Qiu M., Ye C., Zhao X., Zou C., Tang R., Xie J., Liu Y., Hu Y., Hu X., Zhang N. (2024). Succinate exacerbates mastitis in mice via extracellular vesicles derived from the gut microbiota: A potential new mechanism for mastitis. J. Nanobiotechnology.

[17] Zhao C., Hu X., Qiu M., Bao L., Wu K., Meng X., Zhao Y., Feng L., Duan S., He Y. (2023). Sialic acid exacerbates gut dysbiosis-associated mastitis through the microbiota-gut–mammary axis by fueling gut microbiota disruption. Microbiome.

[18] Derakhshani H., Fehr K.B., Sepehri S., Francoz D., De Buck J., Barkema H.W., Plaizier J.C., Khafipour E. (2018). Invited review: Microbiota of the bovine udder: Contributing factors and potential implications for udder health and mastitis susceptibility. J. Dairy Sci..

[19] Wang Y., Nan X., Zhao Y., Jiang L., Wang M., Wang H., Zhang F., Xue F., Hua D., Liu J. (2021). Rumen microbiome structure and metabolites activity in dairy cows with clinical and subclinical mastitis. J. Anim. Sci. Biotechnol..

[20] Zhong Y., Xue M., Liu J. (2018). Composition of Rumen Bacterial Community in Dairy Cows With Different Levels of Somatic Cell Counts. Front. Microbiol..

[21] Vacca M., Celano G., Calabrese F.M., Portincasa P., Gobbetti M., De Angelis M. (2020). The Controversial Role of Human Gut Lachnospiraceae. Microorganisms.

[22] Ma Y., Hu C., Zhang J., Xu C., Ma L., Chang Y., Hussain M.A., Ma J., Hou J., Jiang Z. (2024). *Lactobacillus plantarum* 69-2 combined with α-lactalbumin hydrolysate alleviates DSS-induced ulcerative colitis through the TLR4/NF-κB inflammatory pathway and the gut microbiota in mice. Food Funct..

[23] Liu Y., Liu G., Fang J. (2024). Progress on the mechanisms of *Lactobacillus plantarum* to improve intestinal barrier function in ulcerative colitis. J. Nutr. Biochem..

[24] Wang Y., Li X., Han Z., Meng M., Shi X., Wang L., Chen M., Chang G., Shen X. (2023). iE-DAP Induced Inflammatory Response and Tight Junction Disruption in Bovine Mammary Epithelial Cells via NOD1-Dependent NF-κB and MLCK Signaling Pathway. Int. J. Mol. Sci..

[25] Wellnitz O., Bruckmaier R.M. (2021). Invited review: The role of the blood-milk barrier and its manipulation for the efficacy of the mammary immune response and milk production. J. Dairy Sci..

[26] Yang S., Fang Z., Duan H., Dong W., Xiao L. (2024). Ginsenoside Rg1 Alleviates Blood-Milk Barrier Disruption in Subclinical Bovine Mastitis by Regulating Oxidative Stress-Induced Excessive Autophagy. Antioxidants.

[27] Wellnitz O., Zbinden C., Huang X., Bruckmaier R.M. (2016). Short communication: Differential loss of bovine mammary epithelial barrier integrity in response to lipopolysaccharide and lipoteichoic acid. J. Dairy Sci..

[28] Horowitz A., Chanez-Paredes S.D., Haest X., Turner J.R. (2023). Paracellular permeability and tight junction regulation in gut health and disease. Nat. Rev. Gastroenterol. Hepatol..

[29] Wang J., Li M., Zhao B., Chang R., Wu W., Zhang H., Usman M., Loor J.J., Xu C. (2025). A Disintegrin and Metalloproteinase 17 Disrupts Bovine Macrophage MER Proto-Oncogene Tyrosine Kinase Integrity to Impede Apoptotic Cell Clearance and Promote Inflammation in Clinical Mastitis. J. Agric. Food Chem..

[30] Ou Q., Huang W., Wang B., Niu L., Li Z., Mao X., Shi S. (2024). Apoptotic Vesicles: Therapeutic Mechanisms and Critical Issues. J. Dent. Res..

[31] Miyagishima K.J., Nadal-Nicolás F.M., Ma W., Li W. (2024). Annexin-V binds subpopulation of immune cells altering its interpretation as an in vivo biomarker for apoptosis in the retina. Int. J. Biol. Sci..

[32] Kumar R., Saneja A., Panda A.K. (2021). An Annexin V-FITC-Propidium Iodide-Based Method for Detecting Apoptosis in a Non-Small Cell Lung Cancer Cell Line. Methods Mol. Biol..

[33] Crowley L.C., Marfell B.J., Scott A.P., Waterhouse N.J. (2016). Quantitation of Apoptosis and Necrosis by Annexin V Binding, Propidium Iodide Uptake, and Flow Cytometry. Cold Spring Harb. Protoc..

[34] Wu Y., Hu A., Shu X., Huang W., Zhang R., Xu Y., Yang C. (2023). *Lactobacillus plantarum* postbiotics trigger AMPK-dependent autophagy to suppress Salmonella intracellular infection and NLRP3 inflammasome activation. J. Cell. Physiol..

[35] Shen S., Rui Y., Wang Y., Su J., Yu X.F. (2023). SARS-CoV-2, HIV, and HPV: Convergent evolution of selective regulation of cGAS-STING signaling. J. Med. Virol..

[36] Hopfner K.P., Hornung V. (2020). Molecular mechanisms and cellular functions of cGAS-STING signalling. Nat. Rev. Mol. Cell Biol..

[37] Tan R.T.H., Abdul Rasid S.Z., Wan Ismail W.K., Tobechan J., Tan E.T.Y., Yusof A.N., Low J.H. (2022). Willingness to Pay for National Health Insurance: A Contingent Valuation Study Among Patients Visiting Public Hospitals in Melaka, Malaysia. Appl. Health Econ. Health Policy.

[38] Riley J.S., Quarato G., Cloix C., Lopez J., O’Prey J., Pearson M., Chapman J., Sesaki H., Carlin L.M., Passos J.F. (2018). Mitochondrial inner membrane permeabilisation enables mtDNA release during apoptosis. EMBO J..

[39] Cosentino K., García-Sáez A.J. (2017). Bax and Bak Pores: Are We Closing the Circle?. Trends Cell Biol..

[40] Chattopadhyay S., Marques J.T., Yamashita M., Peters K.L., Smith K., Desai A., Williams B.R., Sen G.C. (2010). Viral apoptosis is induced by IRF-3-mediated activation of Bax. EMBO J..

