# Supplementary material for: Lactobacillus plantarum 17-5 Alleviates Escherichia coli Mastitis by Inhibiting the cGAS-STING Pathway
Source: Animals (Basel). 2025 Nov 16;15(22):3305. doi: 10.3390/ani15223305 (PMC12649193; doi:10.3390/ani15223305)
Supplement: Supplementary file 1 [file animals-15-03305-s001.zip › table s1.pdf]

Supplementary data

Table S1 Antibody Information

Antibody	Manufacture	Catalog Number	Dilution	Source
Bcl2	Wanleibio	WL01556	WB 1:500	Rabbit
Cleaved-Caspase3	Wanleibio	WL02117	WB 1:600	Rabbit
Bax	Wanleibio	WL01637	WB 1:1000	Rabbit
β-Actin	Servicebio	GB15003-100	WB 1:4000	Rabbit
cGAS	ABclonal	A25686	WB 1:1200	Rabbit
STING	ABclonal	A20175	WB 1:700	Rabbit
Phospho-STING	ABclonal	AP1369	WB 1:1000	Rabbit
TANK-binding kinase 1 (TBK1)	ABclonal	A3458	WB 1:1200	Rabbit
Phospho-TBK1	ABclonal	AP1026	WB 1:500	Rabbit
NF-κB P65	ABclonal	A19653	WB 1:5000	Rabbit
Phospho-NF-κB P65	ABclonal	AP0124	WB 1:2000	Rabbit
IκBα	ABclonal	A19714	WB 1:1000	Rabbit
Phospho-IκBα	ABclonal	AP0707	WB 1:500	Rabbit
ZO-1	ABclonal	A25202	WB 1:500; IF 1:50	Rabbit
Occludin	ABclonal	A25320	WB 1:500; IF 1:50	Rabbit
CLDN-3	ABclonal	A2946	WB 1:500; IF 1:50	Rabbit
Secondary antibodies for WB	Servicebio	GB23303	1:10000	Rabbit
Secondary antibodies for IF	Servicebio	GB21303	1:300	Rabbit
