# Supplementary material for: Lactobacillus plantarum 17-5 Alleviates Escherichia coli Mastitis by Inhibiting the cGAS-STING Pathway
Source: Animals (Basel). 2025 Nov 16;15(22):3305. doi: 10.3390/ani15223305 (PMC12649193; doi:10.3390/ani15223305)
Supplement: Supplementary file 1 [file animals-15-03305-s001.zip › table s2.pdf]

Supplementary data

Table S2 Sequences of primer used for RT-qPCR

Gene	Primer sequence (5′-3′)	GenBank accession no.
IL-1β	cct cgg ttc cat ggg aga tg	NM_174093.1
	agg cac tgt tcc tca gct tc	
IL-6	tga aag cag caa gga gac act	NM_173923.2
	tga ttg aac cca gat tgg aag c	
TNF-α	acg ggc ttt acc tca tct act c	XM_005223596
	gct ctt gat ggc aga cag g	
Bax	ctt ttg ctt cag ggt ttc a	XM_015458140
	gct cag ctt ctt ggt gga t	
Bcl2	cat gtg tgt gga gag cgt ca	NM_001166486.1
	tac agc tcc aca aag gcg tc	
Caspase3	ccg agg agg aga cag gat gc	XM_010820245
	cag gcc atg cca gta ttt tcg	
GAPDH	cac cct caa gat tgt cag ca	NM_001034034.2
	ggt cat aag tcc ctc cac ga	
